# Biology-Informed Textile Design: A Mixed-Method Study of Textile Professionals in the UK

**DOI:** 10.1080/20511787.2025.2523620

**Published:** 2025-06-30

**Authors:** Veronika Kapsali, Emmanuel Sirimal Silva, Cathryn Anneka Hall

**Keywords:** sustainability, biologically informed design, textile design practice, NetZero, circular economy

## Abstract

The intersection of the fashion and textile sectors with biological disciplines is seen as presenting opportunities for alternative approaches to industry practices, enabling a transition to NetZero. This study aims to understand the motivations, practices, and barriers faced by contemporary textile designers interested in or actively engaged in this field. We employ a mixed-methods approach to gather qualitative and quantitative data through an online survey of professional textile designers. Thematic analysis and statistical methods are utilized to analyse the respective datasets, and findings are correlated using mixed-methods analysis and information visualization techniques. The findings reveal a paradox: textile designers are motivated to engage with biological disciplines driven by a strong commitment to improving sustainability within the fashion and textile industry, yet their primary focus remains on enhancing the aesthetic qualities of textile products. The study highlights key barriers, such as limited access to specialist knowledge and financial constraints, which hinder the deeper integration of biological principles into broader design practices.

## Introduction

The urgency to reduce greenhouse gas emissions was a key message from COP26 (Wang et al. 2023). However, conclusions drawn from COP27 indicate that commitments to achieve global Net Zero by 2050 fall short of meeting the medium- and long-term goals outlined in the 2015 Paris Agreement (Calster and Reins, 2021). ‘NetZero’ refers to the balance between the amount of greenhouse gases (GHGs) emitted into the atmosphere and the amount removed or offset. Achieving NetZero means that a country, organization, or individual is not contributing to the overall increase of GHGs in the atmosphere. At industrial scale, numerous factors including the complex nature of globalized manufacturing value and supply chains serve as a significant obstacle to implementing and evaluating new NetZero approaches (Bistline, 2021; Renné, 2022; Singh et al., 2023; Vimal et al., 2022). The fashion and textile (FT) sectors exemplify this complexity, as they encompass intricate supply chains that commence with agriculture and/or petrochemical production (raw materials) and involve convoluted manufacturing processes, logistics, and retail. It is noteworthy that the FT industry accounts for approximately 8-10% of annual global CO2 emissions (Niinimäki 2017), ranking it among the most environmentally harmful sectors.

In pursuit of sustainable and innovative solutions, commercial, academic, and third-sector organizations have drawn (Wang et al. 2023) upon the intersection of ecology, biology, and systems theory, seeking a new paradigm for change based on the advice of key texts such as: Natural Capitalism (Lovins, Lovins, and Hawken 2005), Cradle to Cradle (Braungart and McDonough 2009) and Industrial Ecology (Jelinski et al. 1992) which have shaped government policy and industrial strategies in relation to economic, social and environmental sustainability. However, these texts do not offer clear and practical solutions for industrial sectors such as fashion and textiles especially in terms of environmental impacts (Kapsali and Hall 2022) pertinent to this study. In doing so, a gap has been created between the environmental ambitions envisioned by key industrial actors pursuing a transition to circular, regenerative, and sustainable models, and the commercial-scale implementation of novel concepts informed by biology.

The systematic application of principles from biology to engineering design, first pioneered by Otto Schmitt in the 1920s (Harkness 2004), laid the groundwork for these interdisciplinary approaches. Various terms such as biomimetics, biomimicry, biodesign, bionics etc. have emerged to describe innovations resulting from this approach within science, technology and engineering. We adopt the terminology “Biologically Informed (Bio-informed) Discipline” (Iouguina et al. 2014) to define the permeation of this approach beyond the natural sciences and engineering into other academic domains such as social science, art and the humanities. Additionally, we introduce the term “Biologically Informed Textiles (BIT)” to define novel textile design practices informed by biological sciences.

The FT sector already recognizes the potential of biology, either through the direct development of new bio-based materials or indirectly through the introduction of system-based frameworks like the circular economy and regenerative design models, in supporting the attainment of NetZero targets (Byrne 2022). Design practitioners such as Suzanne Lee (Lee et al. 2005) have pioneered the integration of biological principles into textile practice for over two decades, in doing so have inspired a generation of textile practitioners swapping the design studio for a biotechnology lab. Although this has garnered increasing attention, it is not without challenges. This paper presents foundational work that explores the role, opportunities, and challenges of learning and implementing ideas from biology into textile practice. The study is guided by the following research questions (RQ):

RQ1: Why do designers seek ideas from biology to inform their practice?
RQ2: How do textile designers process information from biology and implement it into their practice?
RQ3: What are the barriers preventing textile designers from accessing and implementing information from biology?

## Methodology

We adopted a mixed-methods approach (Johnson, Onwuegbuzie, and Turner 2007; Plano Clark 2017; Creswell 2021), utilizing a questionnaire to capture the attitudes and opinions of professionals in the UK textile design sector. The survey was conducted between April and May 2021, specifically targeting participants within the UK. It was designed to gather insights into the needs, preferences, and challenges faced by the textile design community. This methodology section outlines the key aspects of the survey’s design, data collection, and analysis.

The survey design consists of a series of close-ended questions presented as multiple-choice or multiple response questions. An open-ended option, ‘Other,’ was included within the multiple-choice answers, allowing respondents to provide free-text responses and generate qualitative data. As such, we have two distinct datasets emerging from the survey; quantitative and qualitative.

Relying on a convenience sampling strategy (Silva and Bonetti 2021; Vecchi, Silva, and Jimenez Angel 2021; Demyanova et al. 2023), the survey took place entirely online using the AIRTABLE TM platform to collect and store respondent data. We drew on the professional networks of project partners, Craft Council (UK), Design and Technology Association (UK), as well as the team’s own social media platforms such as LinkedIn and Instagram to recruit participants *via* an open call.

To safeguard the interests and anonymity of survey participants, the survey adhered to ethical guidelines^1^. Respondents’ consent was secured before commencing the survey, and all data collected were stored securely and used solely for research purposes. Any personal identifying information was removed during the data analysis phase to ensure confidentiality.

The survey initiated by prompting respondents to identify their sector within the textile industries, followed by a selection of perceived important aspects of textile design. To ensure participant relevance to the study, we asked if their practice drew on biology in its broadest sense. For participants responding negatively, the survey concluded at this juncture. Affirmative responses directed participants to a query offering various options to elucidate how they applied biological ideas in their textile designs. Additional inquiries explored participants’ motivations for drawing inspiration from nature, methods of discovering biological ideas, and approaches to further investigating these concepts.

The questionnaire concluded with a question related to the barriers, as participants perceived them, which hindered the incorporation of ideas from biology into their practice. To prevent coercion into options incongruent with their views, we introduced multiple response items such as ‘none of the above’ and ‘other’. These options link to free text responses, which also provided respondents with the opportunity to articulate their individual views. The reliability and validity of the responses were considered throughout the design stage by ensuring that technical terms were presented with examples. The questionnaire underwent a review process to eliminate any ambiguous, double-barrelled, loaded, or leading questions by incorporating face validity (Connell et al. 2018) and conducting a pilot test (Atchley, Strayer, and Atchley 2012).

The quantitative dataset, extracted from the multiple-choice responses, was subjected to statistical analysis to extract patterns, trends, and correlations. The raw data was extracted from the AIRTABLE TM platform as a ‘.txt’ file and survey responses were cleaned for inconsistencies and incomplete entries were removed. We used descriptive statistics, i.e. mean, average and percent, to summarize the general trends in participants’ responses. This was particularly useful for questions related to motivations for engaging with biology, preferred sources of knowledge, and barriers faced. Frequency distributions were calculated to understand the prevalence of specific barriers or motivations among the respondents. Finally, results were visualized using charts and graphs to provide an overview of key findings.

The qualitative data, which comprised the free-text entries were systematically reviewed, and initial codes were generated to capture key concepts. These codes were grouped into overarching themes (e.g. “Motivation,” “Access to Knowledge,” “Barriers”) and sub-themes (e.g. “Personal Beliefs,” “Peer-to-Peer Networks,” “Technical Challenges”). This process was conducted iteratively, refining codes and themes as new insights emerged. Information visualization (Onwuegbuzie and Dickinson 2008; Creswell and Clark 2017) was used to map results, with key points from the quantitative data identified as ‘themes’ and ‘sub-themes’ when they converged with the themes from the qualitative data.

Certain limitations should be acknowledged. Firstly, the online nature of the survey may introduce selection bias, favouring individuals with greater access to digital platforms. Secondly, the use of self-reported data may be subject to respondent bias. Lastly, the scope of the survey may limit the depth of insights captured, and additional research may be necessary to explore specific aspects further.

## Results

A total of 104 responses were received out of which 7 respondents indicated that biology did not inspire their textile designs. For this reason, and as they only account for 0.07% of total responses, we decided to remove them from the analysis. This left us with 97 usable responses for the formal quantitative data analysis.

### Sample Description

**Figure 1.**
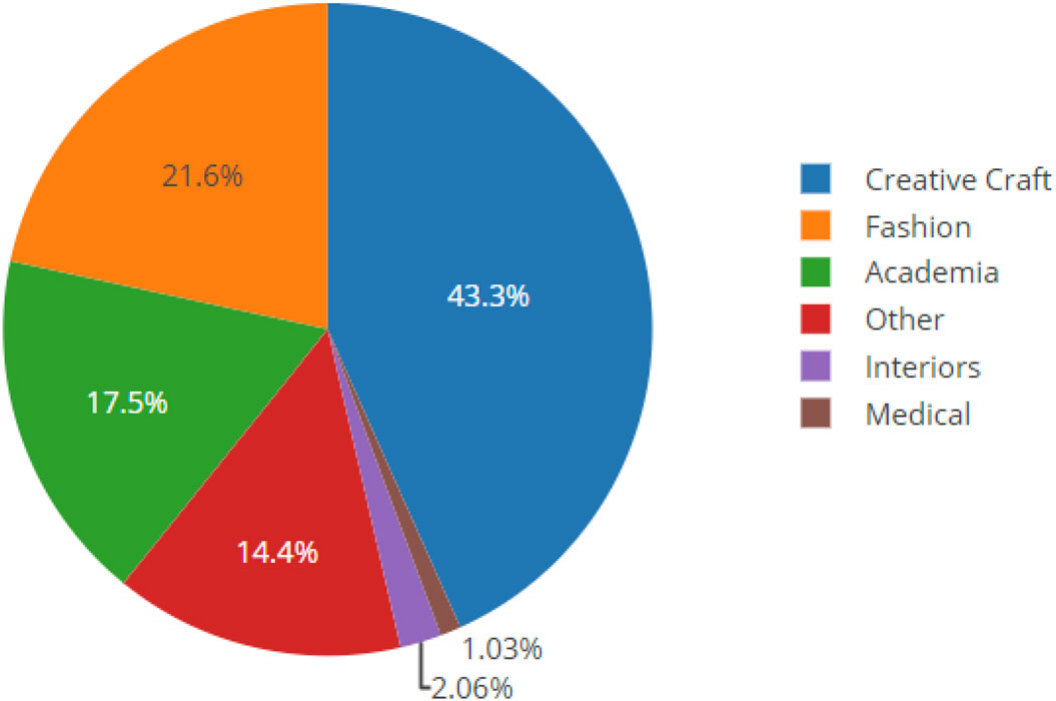
Distribution of sample by textile sector.

Figure 1 summarises the distribution of respondents. The majority (43%) were from the creative craft sector, followed by fashion (22%) and academia (18%). All 97 respondents indicated that biology influenced their textile design practice.

#### Motivation for Seeking Ideas from Biology

This question was presented as a multiple-choice format, allowing participants to select multiple answers (votes). A total of 198 votes were cast in response, with a summary presented in Table 1. The most popular motivation for drawing ideas from biology was environmental concerns, specifically related to the impact of the fashion and textile sector. Notably, 86.6% of the 97 respondents selected this as one of their options.

**Table 1.** Motivations for drawing ideas from biology.

Motivation	Percent of Cases
Environmental considerations	86.6%
Personal beliefs	55.7%
Novelty	36.1%
Other	25.8%

#### Thematic Analysis of ‘Other’ Free Text Responses

Analysis of free-text data is presented in Table 2; we include excerpts from the responses and subsequent coding. We found that the resulting themes from this process align to the responses provided in the multiple-choice element of the survey design but provide some additional insights to these topics.

**Table 2.** Summary of thematic analysis of free text responses.

Exert	Code	Theme
‘Natural colours from food waste’	Sustainability	Environmental
‘To create ‘bio-inspired’ process’	and Recycling	Considerations
‘To create Bio-coatings’		
‘…adding smells…’; ‘…exploration of movement…’;’Multiple properties and applications’; ‘… the process of growth and wear…’	Introduce new Properties	Novelty
‘…use nature directly in my work’; ‘… dyeing with bacteria…’; ‘…using naturally conductive sources.’	Direct Application of Bio-	Novelty
‘From a ‘philosophical’ point of view…. they have a soul of their own’ …. I attempt to sense what plants might want from the textiles…	Philosophical Approach	Personal Beliefs

Table 2 suggests that environmental considerations can include efforts to discover sustainable sources of raw materials originating from biological systems that can be recycled. This aligns to the broader agenda’s driven by the United Nations Sustainable Development Goals (United Nations Environment Programme 2020), the Ellen McArthur Foundation (MacArthur 2013), and European Commission (European Environment Agency 2019) demonstrating the impact of efforts to stimulate new thinking and design cultures among the textile sector, of which the sample of our study is part of.

There also appears to be a strong desire to introduce novelty and newness into textile systems *via* bio-related properties such as fluid movement, multi-functionality, and self-repair. These are achieved through multidisciplinary techniques, including microbiological approaches involving direct interactions with bacteria and enzymes.

Beyond the technical aspects, the study reveals a broader belief system that underpins these endeavours. We note, from the sample, a reverence for nature and a desire to establish a meaningful connection and engagement with the natural environment *via* textile design practice.

It is important to note that although the terms *nature* and *biology* are closely related, they refer to distinct concepts. Nature encompasses the entire physical world, including both living and non-living entities such as animals, plants, mountains, rivers, and atmospheric conditions. Biology, on the other hand, is a scientific discipline specifically focused on the study of life and living organisms. While nature includes all aspects of the natural world, biology is concerned with understanding the life processes within it. However, we begin to observe from the free text responses an interchangeable use of these two terms, which could blur the distinction between the broader scope of nature and the more specialized focus of biology.

### Finding Ideas from Biology

We used a multiple-choice format, allowing participants to select multiple answers (votes). Table 3 summarises the results on how respondents encounter ideas from biology. In total, 228 votes were cast. Interestingly, respondents demonstrated a strategic approach to research during the initial inspiration phase, including visits to places of interest and targeted internet searches. However, the more serendipitous aspects of the creative process were equally represented, with random encounters in natural environments being just as popular. Conversely, randomly conducting internet searches was the least preferred method among the sample.

**Table 3.** How do respondents come across ideas from biology?

Factor	Percent of Cases
Specific visits to a place of interest	68%
Targeted internet search	68%
Random in natural environment	66%
Random internet search	21.6%
Other	11.3%

#### Thematic Analysis of ‘Other’ Free Text Responses

Results from the analysis of unstructured textual data are presented in Table 4. Thematic categories emerging from this dataset provide additional insights into responses obtained through the structured multiple-choice question. Access to Higher Education Institution (HEI) resources (Theme 1), exposure to the Natural environment (Theme 2), self-motivated practical investigation (Theme 3), harnessing the community knowledge pool (Theme 4).

**Table 4.** Summary of thematic analysis of free text responses.

Exert	Code	Theme
Books …. Symposium/ research events.	Educational Resources	1
…lectures at college…		
Specialist libraries and exhibitions Studies at university		
…being in Nature. Investigations in the lab …asking myself how Nature does it	Hands on Experience	2,3
Podcasts and other media	Media	4
Collaboration with FabLab communities Conversations with scientists Collaboration with local lab	Network and Collaboration	4
‘I have Biology and Geography A levels’ ‘…attend seminars’	Own Knowledge	1

Higher educational institutions emerge as primary sources of knowledge, accessed through libraries, lectures, and research dissemination events. This is not surprising as HEIs are hubs for intellectual (knowledge, skills, etc.) and structural capital (libraries, special collections, labs, specialist equipment) that designers can access during their training as well as broader dissemination events such as conferences, exhibitions, etc. Generally, universities and higher education institutions provide students and alumni with access to certain resources, but the extent of this access and its usage would vary widely depending on the institution and the individual graduate.

Immersive engagement with the natural environment appears to be a common practice among the study’s participants, also evident from the multiple choice votes. This highlights a proclivity for seeking inspiration and ideas from direct experiences and in particular the value of immersion within nature (Atchley, Strayer, and Atchley 2012).

Of particular interest is also the theme of self-motivated practical experimentation as an approach of idea generation and development. Scholars of design processes such as Ingold (2013) describes how designers ‘think-through-making’ (Ingold 2013). These alternative ways of thinking and knowing is what sets designers apart and enables them to create innovative solutions to complex problems (Cross 2006).

The utilization of peer-to-peer networks (Masinde and Graffi 2020) composed of individuals with similar interests, diverse backgrounds and expertise levels, is also noteworthy. This suggests that idea sharing *via* digital media and social media platforms occurs among this sample, again this is not a surprise, the fashion and textile sectors in particular use these media to for self-expression, branding, customer engagement, and product presentation (Nash 2019; Cheung and Choi 2022).

#### Developing Ideas from Biology

We used a multiple choice format for this questions, where participants can cast multiple votes. In total, 300 votes were cast by participants and the results are presented in Table 5. The rapid development of the information age has had a significant impact on the approach respondents take to develop design ideas. Seeking advice directly from biologists or specialist online knowledge repositories like nature.org rank lower than Google searches and Google Scholar. Nevertheless, library resources still are the most preferred source regardless of the influence of the World Wide Web.

**Table 5.** Summary of how respondents investigate ideas from biology beyond initial ideation.

Source	Percent of Cases
Library resources	76.3%
General search engine	61.9%
Journals	56.7%
Advice from biologist	49.5%
Knowledge Repository	46.4%
Other	18.6%

Furthermore, whilst in Table 3 we noticed that random internet searches were the least preferred source of accessing ideas from biology, once ideation has taken place, respondents are then open to using general search engines to conduct further research.

#### Thematic Analysis of Motivation ‘Other’ Free Text Responses

The results from the analysis of unstructured textual data are presented in Table 6. From this we observe that textile designers exhibit a proactive approach in seeking out research dissemination events that align with their specific areas of interest and inquiry. The data highlights an inclination toward sourcing external knowledge, whether from specialists or open-source databases, as part of their information-gathering and research endeavours. The role of fostering and refining expertise through practical application and experiential learning builds on the observations from Table 4 and demonstrates the importance of practice based activity to the sample.

**Table 6.** Summary of thematic analysis of free text responses.

Exert	Code	Theme
Conference/ Symposium,	HEI and FE events	1
Bio Hackathons/ summer schools		
‘Creating prototypes.; ‘Experimenting with materials.; ‘I start with a hands-on approach….’; ‘…looking for old techniques that I can use… ;’Own experience’; ‘…. heritage processes and materials’; ‘Thinking through making’; ‘.experimentation… relevant exhibitions.’	Hands on Experience	3
Jstor ;Practical knowledge sites such as Materiom	Online Resources	2
‘Asking open-source community groups’. ‘Consulting other designers from different areas. ‘Conversations with Landcare members…’ ‘Finding people with my same interest online.’ ‘I have written to experts for insight into work.’ ‘Observing and following similar concepts. ‘Seeking expert advice from makers. ‘Speaking with other people.’ ‘Through collaborations with experts’	Open Source/Community Engagement	4

### Implementation of Ideas from Biology into Practice

Table 7 summarises findings related to the way respondents apply ideas from biology into their textile practice. The response options provided *via* multiple choice and each participant could choose more than one response. In total, respondents cast 326 votes across all options. The implementation of biological ideas into aesthetic qualities (i.e. appearance, surface texture, etc.) was the most popular response, receiving 86.6% of the votes.

**Table 7.** How respondents apply their ideas.

How Ideas Are Applied	Percent of Cases
Overall aesthetic qualities	86.6%
Fiber selection	79.4%
To inform the structure of a textile	60.8%
Material processing	45.4%
Textile properties	34.0%
To inform the structure of a yarn	22.7%
Other	7.2%

**Figure 2.**
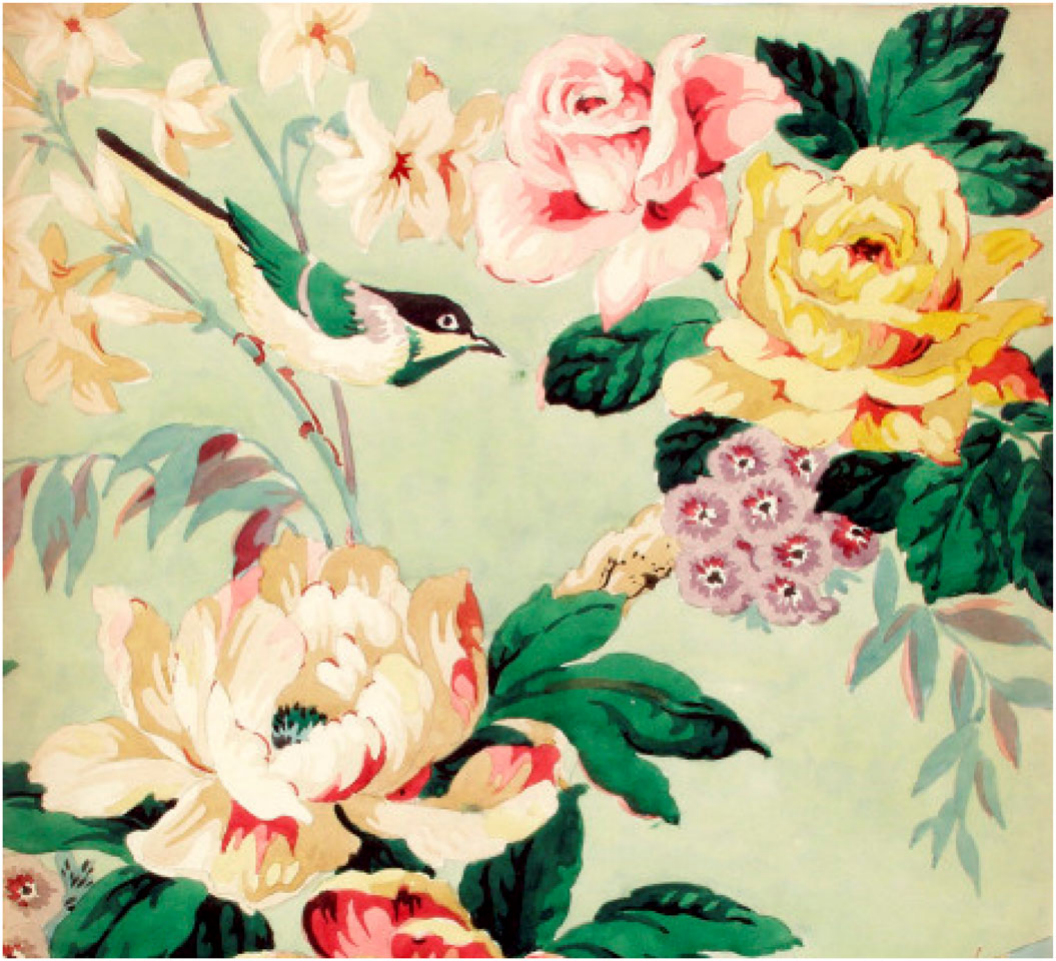
Part of a floral chintz design for a printed textile in watercolour paint, showing rose and rosebud groups with birds, by Lewis Jones, 1936. SD10675. Museum of Domestic Design & Architecture, Middlesex University. Accessed March 04, 2024. https://moda.mdx.ac.uk/object/sd10675/.

This dataset highlights the importance and focus on aesthetics to study participants, this means that the results from research and development activities informed by biology motivate choices related to colour, structure (knitted and woven textiles), texture, surface pattern or a combination of these. This is a very traditional approach in textile design made popular during the industrial revolution, especially direct applications in the form of motifs from the natural environment, such as flowers (Figure 2) and animal skin patterns. This approach requires very little knowledge of biology and is implemented largely *via* observation and drawing skill. Originally, a designer would need to be skilled in hand drawing, but more recently computer aided design has introduced a new set of skills that can be applied to this type of design.

Fibre selection primarily refers to decisions related to the composition of textiles during the raw material sourcing phase. According to our sample, engaging with biology-based topic during the research and development phases directs designers towards natural fibres such as wool or cotton, and man-made fibres that are promoted as ‘sustainable’, meaning that they do don’t come from finite sources such as petrochemicals. Similarly, this aspect requires very little knowledge of biology and is implemented *via* the material selection part of the design process. Here, designers rely on the description provided by the material supplier, For example, organic cotton, or recycled polyester, to attribute environmental impacts to their products. To date, this is hugely problematic because of the lack of transparency across fashion and textiles value chains (McQuillan 2012; Niinimäki 2017; Manshoven et al. 2019; Palm, Cornell, and Häyhä 2021).

Material processing refers to designers engaging with the way the raw materials are manufactured and processed. Often, designers operating in this space have fostered an interest in biotechnology and have developed novel methods for developing their own raw materials using bacteria or other micro-organisms. This mode of practice requires working knowledge of applied microbiology and typically occurs at small scale, on an artisanal or research level. While, at the point of publication, there are few commercial scale developments that have emerged from this space (i.e. Spiber^2^). Nonetheless, there is a strong push from the design community to identify alternative sources of materials for textiles that are sustainable and can fit within a circular system (Cole et al. 2012; European Environment Agency 2019; Lee et al. 2020; United Nations Environment Programme 2020).

#### Thematic Analysis of Motivation ‘Other’ Free Text Responses

The outcomes arising from the analysis of free text data are shown in Table 8. The themes have been assigned numerical identifiers and are categorized as follows:

**Table 8.** Summary of thematic analysis of free text responses.

Exert	Code	Theme
Aesthetic appeal	Aesthetic Appeal	1
‘…awe reaction to its seamless ingenious beauty’		
‘a need of reconnecting with Mother Nature’; ‘…learning & aware of Nature…’; ‘Creating connectedness to nature.’; ‘Inspired by the structures created by nature ‘; ‘separating’ humans from nature ‘ use our 5 senses to draw inspiration.; ‘… environmental survival…’; ‘a sense of oneness with our environment’ ‘I fear we are losing connection with the natural world’	Connecting with nature	2
Anthropological (Identity of Place) contribute to a humanitarian Environment. ‘Efficiency in performance’ ‘Health benefits and wellbeing’ ‘Locally available, use of 5 senses, cost effective.’ Preserving cultural heritage	Environmental and human impact	3

1. Aesthetic
2. Personal belief
3. Impact

Textiles have long served as a conduit for designers to express and communicate their personal perspectives and values. This is particularly evident in the provided sample, where it becomes clear that some designers utilize their textile practice as a platform to articulate their appreciation for the aesthetic appeal of natural specimens and phenomena. The work presents a personal statement of the designer’s worldview as discussed earlier. For some designers participating in this study, this embodies a strong desire to reconnect with the natural environment as well as a sense of responding to the environmental challenges.

### Key Barriers

The results from Table 9 allows us to rank the key barriers preventing the respondents from using biology as a model in their own practice (from most important to least important) as: access to specialist knowledge, access to examples of biologically informed textile designs, opportunities to access sources of inspiration from nature. We found that the most significant barrier is the access to specialist knowledge, which received 79.4% of the votes. This data may shed light on why the option of ‘seeking expert advice from a biologist’, ranked low, as it underscores the potential barriers in accessing specialist knowledge.

**Table 9.** Barriers preventing the use of biology in participants’ own practice.

Barrier	Percent of Cases
Access to specialist knowledge	79.4%
Access to examples of biologically informed textile designs	44.3%
Opportunities to access sources of inspiration from nature	18.6%
Other	21.6%

#### Thematic Analysis of Barriers ‘Other’ Free Text Responses

The results derived from the dataset analysis are displayed in Table 10. Each theme is assigned a numerical identifier as follows:

**Table 10.** Summary of thematic analysis of free text responses.

Exert	Code	Theme
Access to equipment/ lab facilities	Access to equipment	1
Access to lab-based equipment Specialist equipment… … to push my learning and development further		
‘Discussions and conversations with biologists and researchers in the area. ‘Isolations from specialists from different disciplines	Access to specialists	2
‘Financial barriers. I am a small company … ‘Current barriers are almost exclusively financial.’ ‘Lack of funding and opportunities’	Financial constraints	3
‘A lack of scientific background’ ‘Don’t have the foundations in biology’ ‘The lack of knowledge of how things work in nature.’ ‘Difficulty reaching the researchers’ ‘Understanding chemical and molecular side’	Knowledge Gaps	1,2

1. Equipment
2. Knowledge and Skills
3. Funding

Building upon the insights from previous sections, it is unsurprising that participants perceived a lack of access to specialist knowledge and skills as a key obstacle. The free text dataset further highlighted that limited access to physical resources, such as labs and equipment, is another significant hurdle. Additionally, we note that limited resources in the form of financial support restrict opportunities for practitioners to engage in this type of activity.

## Discussion

The aim of this study is to deepen our understanding of biological disciplines as a source of knowledge and ideas for textile design practitioners and set a benchmark to measure future developments. In this section we will review the findings within the context of the three research questions which have underpinned the study from the onset.

**Figure 3.**
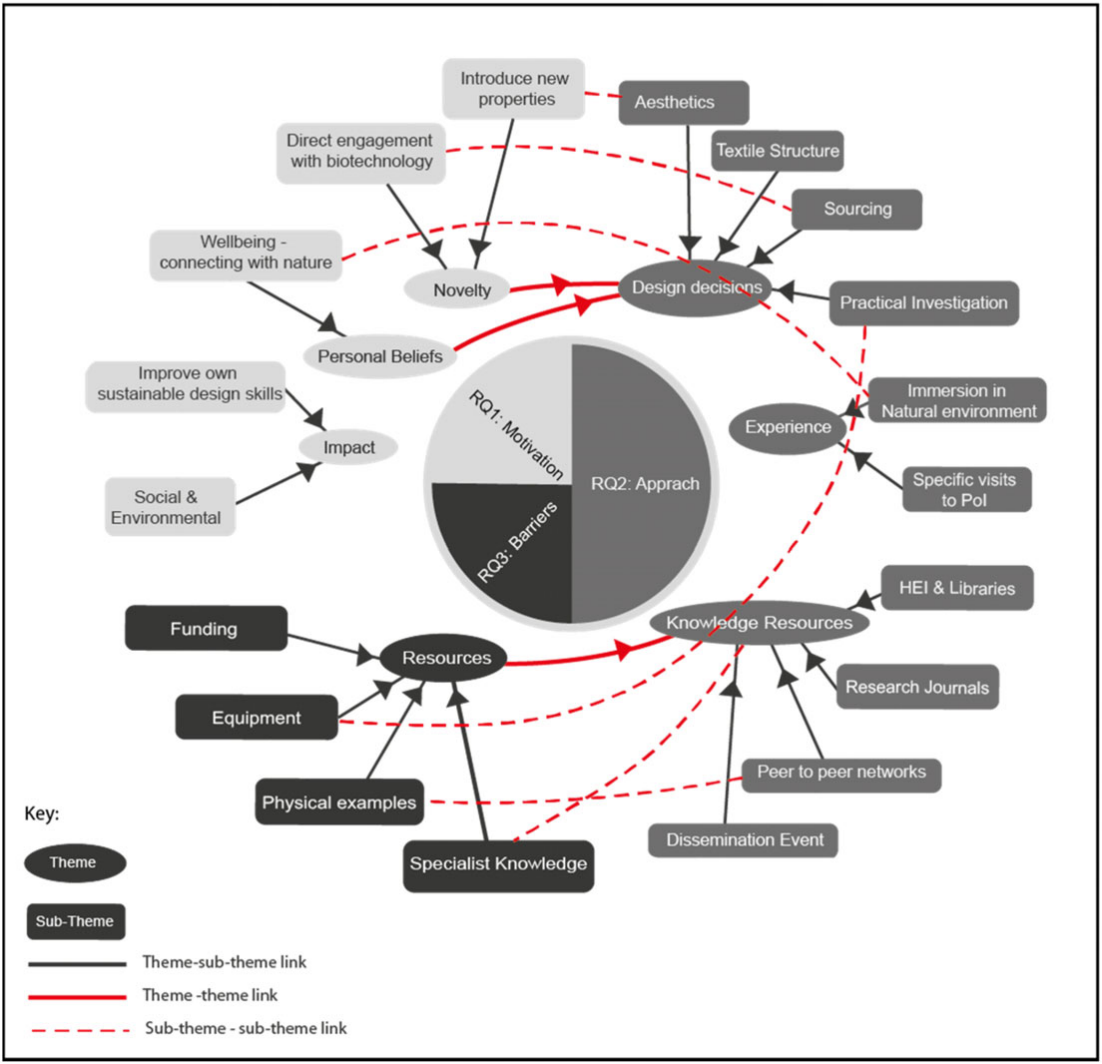
Summative map illustrating the relationships between themes and sub-themes across statistical and free text data.

To guide the discussion we present Figure 3 which integrates both qualitative and quantitative data, highlighting the emerging themes and sub-themes from the analysis of quantitative and qualitative data in relation to the study’s three research questions: Motivation (RQ1), Approach (RQ2), and Barriers (RQ3). Each of these research questions is positioned at the centre of the diagram, with broader themes and more specific sub-themes radiating outward.

Connections between themes and sub-themes are illustrated with solid lines, showing how each sub-theme supports or relates to its parent theme. In addition, red dashed lines highlight significant connections between sub-themes, indicating areas where concepts overlap or interact across different themes. For example, the sub-theme “Novelty” links with both “Personal Beliefs” and “Design Decisions,” demonstrating the interconnectedness of innovation and decision-making in the context of textile design.

It is important to note that this schematic does not incorporate any quantitative data weighting, ensuring an equal representation of themes and sub-themes based purely on their qualitative importance within the study’s findings. The diagram serves as a visual tool for understanding how key insights from the study are interrelated and aligned with the research questions.

### Why Do Textile Designers Seek Ideas from Biology (RQ1)?

Concerns relating to the environmental impacts of the fashion and textile industries are at the forefront of most respondents’ minds. This is coupled with a desire to contribute to the discourse around addressing these challenges through their creative practices. Lerpiniere (Lerpiniere 2020) highlights that textile designers, with their expertise in fibres and fabrics, are uniquely equipped to address sustainability issues in the fashion and textile sectors. Testimony to this trend is the increasing number of fashion and textile designers and researchers who are dedicating their work to identifying alternative, sustainable sources of raw materials and exploring pathways to transition to a circular economy (Kane and Philpott 2013; Kate Goldsworthy and Politowicz 2018; Forst 2020; Ribul, Goldsworthy, and Collet 2021).

From the analysis of the open-ended data, we learned that personal belief systems play a significant role in driving design practice. Personal belief systems, within the context of this study, refer to interconnected beliefs, values, and attitudes that shape an individual’s interpretation and navigation of the world. Participants share a reverence for the natural world, which manifests as a deep respect and admiration. This reverence is not just a sentiment specific to textile designers but is also recognized within the broader creative sector (Atchley, Strayer, and Atchley 2012). The findings highlight the influence of this view on the values and practices of participants, circling back to the previous point on participant motivation to reduce the sector’s environmental impact and promote eco-friendly practices.

While this driver is not surprising, the analysis reveals a deep-seated desire among survey participants to establish a meaningful connection with the natural world. Notions such as *losing touch* and *becoming disconnected* from nature, along with its impacts on general mental health and wellbeing, were unexpected. To study these insights further requires specialist knowledge from the field of Environmental Psychology which is outside the scope of the present study.

### How Do Designers Identify, Develop, and Implement Ideas from Biology (RQ2)?

From our findings, *access to knowledge* and *experience* emerge as key factors in the development and implementation of ideas from biology. Observations (see Table 4) highlight that higher educational institutions emerge as pivotal sources of knowledge, accessed through libraries, lectures, and research dissemination events. Surprisingly, despite the digital age, physical resources and experiences remain the preferred source of information for participants.

Experience plays a pivotal role not only in sparking inspiration but also in the evolution of ideas. A recurring theme that emerges is self-motivated practical experimentation, where designers actively engage in the synthesis and application of biological concepts through hands-on experimentation. Practice-based research methods and experimentation form the backbone of the design process (Cross 2006). Within the realm of textiles, Kane and Philpot (Kane and Philpott 2013) highlight that hand-making and craftsmanship are integral processes employed by textile practitioners to develop a comprehensive understanding of both materiality and concept. The dataset underscores the recurring theme of nurturing and honing knowledge and expertise through the practical application of skills and experiential learning.

The findings also highlight that peer-to-peer networks comprising individuals with diverse levels of knowledge and expertise, serve as valuable resources for idea generation and concept development. Insights from Table 6 indicate that some textile designers take a proactive approach in seeking research dissemination events aligned to their specific interests. This highlights designers’ inclination to source information from biology, whether from specialists, peer groups or open-source databases, as part of their information-gathering and research endeavours.

The most prevalent approach to the implementation of ideas from biology is within aesthetic qualities (86.6%). Igoe (2013) explains that textile designers often go beyond the aesthetic qualities whilst designing, for example “fabric structure determines the dyes used, the thickness of the printed mark permissible for a good finish and the density of colour achievable” (ibid, p.102) which demonstrates how technical and tacit textile knowledge are honed to deliver specific aesthetic qualities. A notion extended by Lerpinere’s (2020) views on the potential of this knowledge for enhancing sustainable practices within the fashion and textiles sectors. Although Igoe’s position explains the tendency to interpret and develop ideas using traditional approaches such as visual studies to inform designs and then apply technical knowledge to implement these, there seems to be a disconnect with Lerpiner’s position which highlights that designers are not necessarily make full use of the range and scope of information emerging from these emerging interdisciplinary spaces (i.e. biology and engineering).

Here we find a paradox: textile designers participating in this study demonstrate strong motivation to improve industry practices and overall sustainability of the fashion and textile sector by looking at the emerging interdisciplinary spaces that intersect biology and textile design, guided by global sustainable, circular economy agendas. While we have emphasized that textile designers are well-positioned to address sustainability challenges, the precise mechanisms for achieving this through a bio-informed approach remain elusive.

Despite endorsements from influential organizations and thought leaders, the impact of insights drawn from biological disciplines seems disproportionately focused, in practice, on the aesthetic qualities of textile outputs. While this emphasis is significant, it may fall short of the industry transformation that these designers aspire to achieve. In summary, the intersection of sustainability, biology, and textile design presents both promise and ambiguity—a paradox that invites further exploration and innovation.

### What Are the Barriers Preventing Textile Designers from Engaging with Biology Disciplines (RQ3)?

Bio-inspired design (BID) is a relatively established concept within engineering education, especially as it offers opportunities to cultivate essential competencies among engineering students and assist engineers in developing the ability to collaborate across disciplines, which is crucial for effectively addressing complex challenges (Nagel et al. 2019; Hashemi Farzaneh 2020). However, the adoption of BID methods in academic and industrial settings by engineers has been limited (Glier et al. 2011; Barthlott, Rafiqpoor, and Erdelen 2016; Vandevenne, Pieters, and Duflou 2016), primarily due to challenges in understanding biological solutions and transferring these analogies to develop technical solutions (Nagel, Schmidt, and Born 2018; Chen et al. 2023). These challenges are further compounded by the complexity of biological systems and the difficulty in dealing with unstructured data (Fu et al. 2014).

For textile designers participating in this study, the central challenge lies in accessing specialized knowledge. While respondents can tap into resources such as libraries, journals, and conferences, these outlets predominantly cater to an audience versed in STEM disciplines (science, technology, engineering, and math) who also find BID challenging. As such, initial obstacles such as culture, language and specialised forms of knowledge, further compound the difficulty for textile designers seeking to engage with this subject. Bridging this gap requires targeted efforts to make bio-informed insights more accessible and comprehensible to the diverse community of textile designers.

The open-text responses shed light on additional obstacles extending beyond access to specialist knowledge and essential skills to securing equipment and financial support for developmental initiatives. Addressing these challenges necessitates strategic efforts to bolster funding mechanisms and foster a multidisciplinary environment for textile innovation.

### Limitations of This Study

While this study provides insights into the motivations, practices, and challenges of textile designers seeking inspiration from biology, it is important to acknowledge its limitations. Firstly, the research relied on self-reported data from a survey, which may be subject to response bias. Participants may have provided responses that reflect their perceived motivations and practices rather than their actual behaviours. Secondly, the sample size and demographic composition of the survey respondents may not fully represent the diversity of textile designers worldwide. The study primarily focused on a specific group of designers, and the findings may not be generalizable to designers from diverse cultural backgrounds or levels of experience. Additionally, this study used quantitative methods for data collection and analysis, with limited qualitative exploration. A more in-depth qualitative approach, such as interviews or focus groups, could offer richer insights into the intricate nuances of designers’ motivations and practices.

Furthermore, the study primarily focused on the perspectives of textile designers themselves, without extensive input from biologists or other stakeholders in the field. A more comprehensive examination involving multiple perspectives could provide a more holistic understanding of the dynamics between biology and textile design.

Lastly, the study examined the current situation within the industry, and the findings may not account for potential future developments in textile design or advancements in biological sciences that could influence designers’ practices and motivations. These limitations should be considered when interpreting the results and conclusions of this study, and future research endeavours may seek to address these constraints for a more comprehensive understanding of the subject matter.

## Conclusion

This study set out to explore how biological disciplines can serve as a source of knowledge and inspiration for textile design practitioners. Through a mixed-methods approach, combining both qualitative and quantitative data, we investigated the motivations, approaches, and barriers that shape how designers engage with biology-informed practices. Our findings provide a detailed understanding of these dynamics, mapping the relationships between key themes related to motivation (RQ1), approach (RQ2), and barriers (RQ3) across the textile design sector.

The research underscores the significant role environmental concerns play in motivating textile designers to seek inspiration from biology. Many participants expressed a strong commitment to sustainability, driven not only by practical concerns but also by personal belief systems and a desire to reconnect with nature. This emotional and philosophical connection reveals a deeper layer of motivation, with designers viewing their work as an opportunity to both mitigate environmental impact and enhance personal well-being. This growing reverence for the natural world reflects broader cultural shifts within the design community, where responsibility for environmental stewardship is increasingly intertwined with creative practice.

When examining how designers identify and implement biological ideas, our findings reveal that access to knowledge and hands-on experience are crucial. Higher education institutions, peer networks, and physical experimentation remain pivotal in facilitating the application of biological concepts. However, despite these resources, the use of biology in textile design is often limited to aesthetic qualities, rather than more systemic applications. This narrow focus suggests a gap in fully harnessing the breadth of interdisciplinary knowledge available, particularly in areas like material innovation and sustainability.

The study also highlights significant barriers preventing more comprehensive adoption of biology-informed practices. Many textile designers, particularly those without a STEM background, face challenges in accessing and understanding the complex knowledge emerging from biological disciplines. This gap is further complicated by the lack of specialised equipment and funding, limiting opportunities for smaller or independent designers to innovate. These barriers point to a broader issue of inclusivity within the textile industry, where access to cutting-edge knowledge and resources is often unevenly distributed.

The deeper implications of these findings suggest that for biology-informed design to reach its full potential, the textile industry must address these barriers. There is a need for stronger interdisciplinary collaboration, particularly between designers and experts in biology, to bridge the knowledge gap and foster innovation. Educational programs should also evolve to integrate biology more comprehensively, equipping future designers with the necessary skills and frameworks to engage with bio-inspired practices. Furthermore, industry-wide policy changes and support structures are essential to scale sustainable practices and make biology-inspired innovation the norm rather than the exception.

Although textile designers are well-positioned to drive the sustainability agenda, their ability to fully integrate biological principles into their work is constrained by limited access to knowledge, resources, and funding. By fostering greater interdisciplinary collaboration and addressing these barriers, the textile industry can unlock the full potential of biology-informed design, leading to more sustainable, innovative, and impactful practices. Future research should continue exploring these intersections, with a particular focus on how to improve knowledge transfer and support the practical application of bio-inspired design, ultimately contributing to a more sustainable, circular, and net-zero textile industry.

## Notes

1 https://www.ukri.org/councils/esrc/guidance-for-applicants/research-ethics-guidance/.

2 https://spiber.inc/en/.
